# 

**Published:** 1881-03

**Authors:** 


Vol. XXXV. **MARCH, 1881.** No. 3.

THE

### Dental Register,

A MONTHLY JOURNAL,

DEVOTED TO THE INTERESTS OF THE PROFESSION.

EDITED BY J. TAFT, D. D. S.

PUBLISHED BY

SPENCER & CROCKER,

OHIO DENTAL DEPOT,

NOS. 117, 119, 121 WEST FIFTH STREET, CINCINNATI, OHIO.

*TERMS—$2.50 Per Annum, in Advance. Single Copies, 25 Cents.*

